# Face Averages Enhance User Recognition for Smartphone Security

**DOI:** 10.1371/journal.pone.0119460

**Published:** 2015-03-25

**Authors:** David J. Robertson, Robin S. S. Kramer, A. Mike Burton

**Affiliations:** 1 School of Psychology, University of Aberdeen, Aberdeen, United Kingdom; 2 Department of Psychology, University of York, York, United Kingdom; Liaoning Normal University, CHINA

## Abstract

Our recognition of familiar faces is excellent, and generalises across viewing conditions. However, unfamiliar face recognition is much poorer. For this reason, automatic face recognition systems might benefit from incorporating the advantages of familiarity. Here we put this to the test using the face verification system available on a popular smartphone (the Samsung Galaxy). In two experiments we tested the recognition performance of the smartphone when it was encoded with an individual’s ‘face-average’ – a representation derived from theories of human face perception. This technique significantly improved performance for both unconstrained celebrity images (Experiment 1) and for real faces (Experiment 2): users could unlock their phones more reliably when the device stored an average of the user’s face than when they stored a single image. This advantage was consistent across a wide variety of everyday viewing conditions. Furthermore, the benefit did not reduce the rejection of imposter faces. This benefit is brought about solely by consideration of suitable representations for automatic face recognition, and we argue that this is just as important as development of matching algorithms themselves. We propose that this representation could significantly improve recognition rates in everyday settings.

## Introduction

Face recognition remains a significant challenge for the cognitive sciences. Our understanding of human face perception extends across a wide range of signals, such as facial expression, speech, eye-gaze and attractiveness [*1*]. Despite this, rather slower progress has been made in understanding the processes involved in recognising a person’s identity [*2,3*]. In computational approaches to face recognition there has been greater progress, and systems now exist which can out-perform *unfamiliar* human observers on some face recognition tasks [*4,5*]. However, it is well-established that human observers are considerably more accurate in recognising familiar than unfamiliar faces, and this holds whether they are asked to remember photos [*6,7*], to match two simultaneously-presented images [*8–10*], or to match a live person to a photo-ID [*11,12*]. To date, no automatic face recognition system approaches the levels of accuracy and generalizability observed in human observers presented with *familiar* faces.

**Fig 1:**
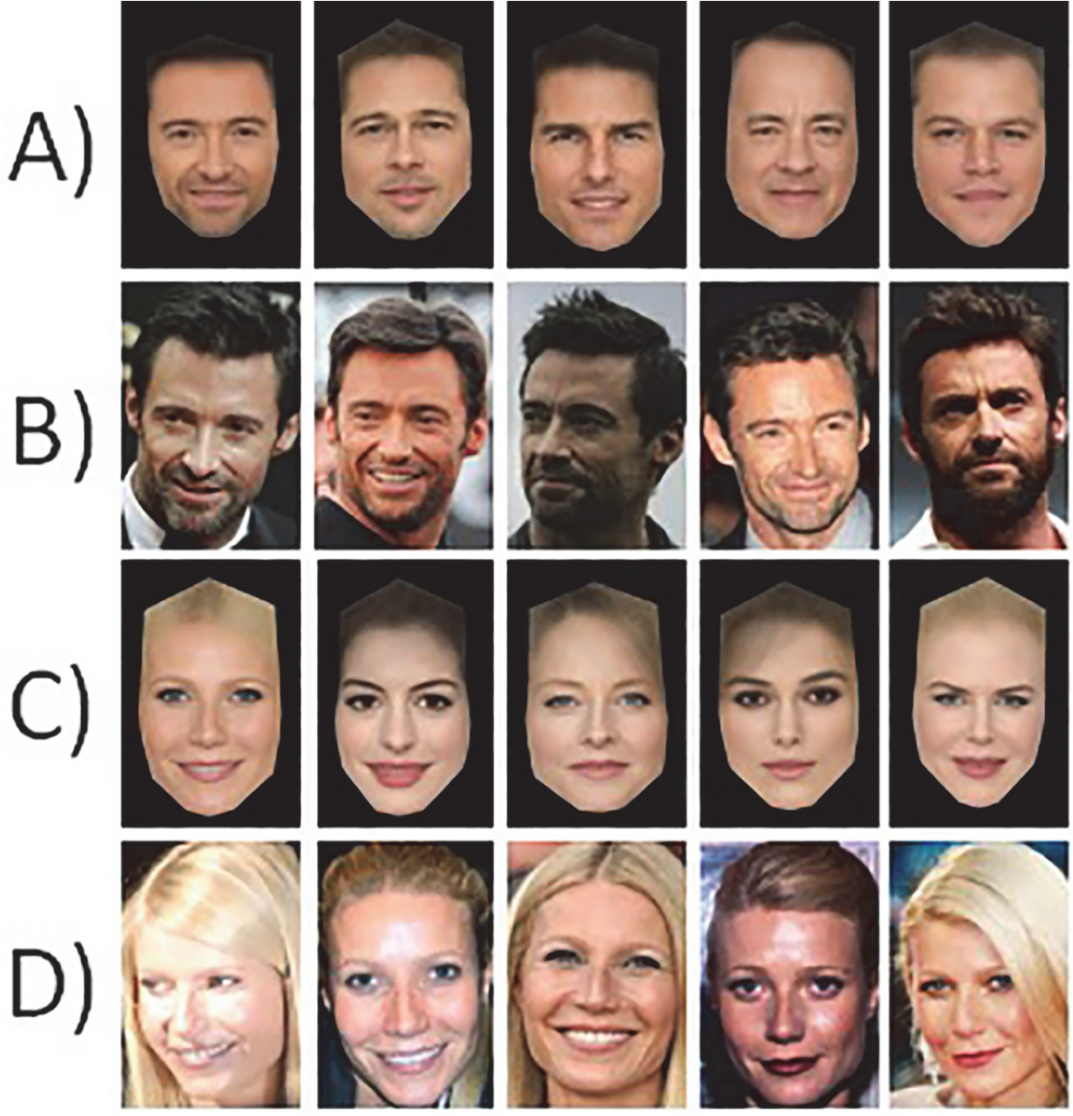
Experiment 1 example stimuli. Rows A and C show celebrity face averages. Rows B and D show examples of individual images. (For copyright reasons, we are not able to present the individual images used in the experiment—which were chosen mechanically on the basis of a selection rule. The individual images shown in rows B and D are legally reproducible, and are a close approximation of the actual stimuli. See S1 File for celebrity names and image licence and attribution information).

If computational approaches to face recognition are to become truly useful, then they should incorporate those aspects of *familiar* face recognition that allow for high levels of performance. How might these be characterised? One key component is that familiar face recognition is able to operate over huge variations in image, within the same person [*13*] (see Fig. 1). A person’s face can vary considerably as a result of changes in lighting, camera characteristics, pose, expression and age. Given this variability, two images of the same person can easily be less similar than two images of different people [*14*]. One way to tackle this variability is to propose that familiar viewers actually store many different images of the same person in memory [*15*]. However, an alternative proposal by Burton, Jenkins, Hancock and White [*16*] is that familiar viewers form an abstract representation that retains aspects of the face that are common across different images, while discarding those aspects which vary. One candidate for such a representation is a facial average, in the sense of morphing together many different images of the same person [*17*].

Facial averages have been shown to be useful in computer-based face recognition [*16*]. In order to improve the performance of automatic recognition systems, developers have typically focused on creating the best image matching algorithm [*18*]. However, employing an average-based approach is independent of the matching technique used. Instead, this approach focuses on what is to be matched. Burton et al [*16*] propose that research on the stored representation itself is just as important as research on matching, and in particular that a focus on representation may provide a route to understanding the impressive levels of accuracy human viewers can achieve with familiar faces.

In a test of the real world utility of face averages, Jenkins and Burton [*19*] assessed the accuracy of an online version of the (then) industry standard recognition system FaceVACS. This system contained a large database of celebrity face photographs (over thirty thousand images, and over three thousand celebrities) which varied considerably in illumination, pose, facial expression, age, and image quality. In response to a probe photo, the system would return the closest matching image in its database. Jenkins and Burton [*19*] tested 20 different images of each of 25 different celebrities, and found that on 54% of occasions, an image of the same celebrity was returned—quite an impressive hit rate, given the number of people in the database. However, when they tested average images, built from each of the 20 images for each person, the hit rate rose to 100%. Performance of 80% was achieved even when the averages were constructed entirely from individual photos that the system had previously failed to recognise.

The findings of Jenkins and Burton [*19*] established the effectiveness of face averaging as a means of improving face recognition in a commercially available algorithm, used for large-scale security applications such as airports. However, automatic face recognition has now reached consumer-level products. In particular, several smartphones currently on the market use faces for security. We store a significant quantity of important personal information on our phones [*20*], and this personal information is at risk of unauthorised access if the device is lost or stolen. Alphanumeric password systems have known vulnerabilities, such as shoulder-surfing [*21*] and analysis of the oily residue from fingers [*22*]. For these reasons, there is growing interest in biometric security, including face recognition.

In the studies presented below, we examine the accuracy of face recognition security software in the currently popular Samsung Galaxy range of smartphones (S3, S4, S5). This is used to unlock phones using the owner’s face, as an alternative to alphanumeric passwords. However, we should note that this is currently marketed as a low-security feature, and can be over-ridden with a password. We should also note that the details of the face recognition algorithm are proprietary, and therefore not open to public examination. Nevertheless, it is interesting to establish how accurate this system actually is. In the following experiments, we test this system using standard face-matching (image to image comparison), and also average-based matching (image to average). In the first experiment, we do this using photos of celebrities, for whom there are many varying images available. In Experiment 2, we test face-matching with phone users, replicating the conditions in which face-based security is normally used with these smartphones. To anticipate the results, we find quite high levels of face recognition, but these are significantly improved by the use of averages. Once again, this supports the proposal that there is much to be gained by focussing on ‘what is to be matched’, as well as the more common approach of studying matching algorithms themselves.

## Experiment 1: Celebrity Images

In this experiment we examined celebrity recognition using a smartphone. Facial security works by storing an image of the user (the ‘target’) for subsequent comparison with images captured during unlocking attempts. Here, we compared recognition performance for a target image comprising a photo (the normal usage) with performance using a target image comprising an average, derived from many photos of the target person.

### Materials and Methods

#### Smartphone Specifications

A Samsung Galaxy S4 smartphone (model GT-19505) with an Android operating system (v.4.4.2) was used in the present experiment. The face authentication security feature is part of the standard software package installed on the device. Throughout the experiment, the phone was placed in ‘flight mode’, a feature which disables all wireless functions while retaining full use of the camera. To capture the live face of the user, the recognition algorithm relies on a front-facing two megapixel camera/video recorder (30 frames/s).

#### Stimuli and Apparatus

Ten different celebrity identities (5 male/5 female) were used in this experiment. For each identity, 35 large colour face photographs were downloaded from Google Images. We chose the first 35 images which met the following criteria: (i) no part of the face should be obscured (e.g. by clothing, glasses, or a hand); (ii) pose should be very broadly full-face in order to allow the placement of facial landmarks; and (iii) pose should be standing or sitting, but not lying down, in order to limit the angle of the head to a relatively upright position. The images varied with respect to environmentally generated parameters (e.g. lighting) and face-based parameters (e.g. pose, facial expression, hair style). All of the selected images were cropped to a size of 380 x 570 pixels.

Individual average faces were derived from the first 30 images of each person (see Fig. 1 for examples). Images were marked up using 82 hand-placed anatomical landmarks. The average ‘shape-free’ texture component was derived by morphing the 30 images to a standard face template and calculating the mean RGB values for each pixel. Each celebrity’s average texture was then morphed onto their average shape to produce their face average (for a more detailed description of the averaging process, see Burton et al [*16*]).

All images were presented on an 18 inch Dell PC Monitor (1440 x 900 screen resolution, 60 Hz refresh rate) using E-Prime 2.0. The smartphone was securely affixed to a chinrest with the front of the device facing the PC screen. A distance of 24.5cm was maintained between the screen and the smartphone during testing; this ensured that each face image would fill the oval face capture space provided by the authentication software.

#### Design and Procedure

Five images of each celebrity were used as test images throughout. These were used to try to unlock the phone that had been encoded with targets that were either (i) a celebrity average, or (ii) a celebrity photo. Average targets were derived from 30 images, not including the five test photos (see above). Individual image targets were five randomly chosen photos from the set of 30 used to construct the average (we used five different individual-photo targets in order to avoid bias which may be induced by chance selection of a particularly good or particularly poor likeness of an individual). Targets were encoded into the phone by photographing images from the computer screen, and the same method was used to test images for unlocking.

Each average celebrity target was tested against the five test items, and against five randomly-selected same-sex test items of other celebrities (‘imposter images’). Similarly, each individual-photo target was tested against these test and imposter images. For each combination, we simply recorded whether or not the phone was unlocked by the test image. In summary each celebrity was tested 60 times as follows: 25 single-image ‘user’ trials (5 individual-image targets x 5 different test images of the same person); 25 single-image ‘imposter’ trials (5 individual-image targets x 5 different test images of different people); 5 average-image ‘user’ trials (average-target x 5 test images of the same person); and 5 average-image ‘imposter’ trials (average-target x 5 test images of different people).

### Results

Mean performance level across the ten celebrities is shown in Table 1, this shows that user recognition is considerably better for average-image targets, than for individual-image targets, and analysis shows that this advantage is significant (Wilcoxon signed-rank test, two-tailed: *W* = 2, *p* < .05). There were no imposter errors, with both types of target giving perfect performance.

**Table 1:** Performance levels for the individual and average-image targets.

	Individual-Image Target	Average-Image Target
User Recognition	45% (22)	68% (37)
Imposter Rejection	100% (0)	100% (0)

*Note*. For each celebrity, individual-image targets were tested 50 times (5 targets x 5 test images for both ‘users’ and ‘imposters’), while average-image targets were tested 10 times (average target x 5 test images for both ‘users’ and ‘imposters’). The table shows mean performance by condition across all celebrities (*SD* in parentheses).

These results show a large benefit of using average-image targets over individual images. This is a promising finding, because it suggests that a change in the stored representation of a user’s face (in this case an average) can bring large performance improvements—a benefit which does not require any change to the underlying matching algorithm. Furthermore, the large benefit in ‘user’ recognition carries no cost in terms of imposter rejection. The smartphone rejected all imposters on this test—whether encoded with individual or average-image representations of the face.

Despite the clear average-advantage in this preliminary experiment, this use of the phone is somewhat unusual. In order to retain control over extraneous variables, this experiment used a lab-based set up, in which all images were derived from photographing a computer screen. In the next experiment, we test the phone in the common setting—where users encode their own target images, and use these to unlock the phone live.

## Experiment 2: Real Faces

In this experiment we test whether the face average advantage reported in Experiment 1 generalises to normal use with real faces. To this end, we encoded the phone either with individual photos or the face average of a participant, who was then asked to unlock the phone using their face. To examine the generalizability of the face recognition system, we conducted recognition trials in six different contexts. We used five indoor locations with variable lighting conditions: an office, a corridor, a staff room, an atrium and the driver’s seat of a stationary car. This manipulation aims to recreate the variety of indoor environmental conditions that a smartphone user would typically encounter. Additionally, in daily life we also require regular access to our phone when outdoors (e.g. reading e-mails while traveling to work). The development of a face recognition algorithm which is as effective outdoors as it is indoors, is one of the major challenges facing developers [*23*], and so here we also test the potential for an average advantage in an outdoor setting (next to an office block on a university campus).

When constructing average target faces for this experiment, we reduced the number of images contributing from 30 (in Experiment 1) to 7. As well as examining any theoretical advantage for average photos over individual photos, we are also interested to establish whether this technique might lead to a realistic improvement in day-to-day use. For this purpose, it seemed too onerous to request participants to take a large number of photos of themselves, and so we chose just seven here. In line with the findings from Experiment 1, we predict that real face recognition rates will be greater when the stored target is a face average, rather than an individual photo.

### Materials and Methods

#### Ethics Statement

This study was approved by the Ethics Committee of the School of Psychology, University of Aberdeen, acting under the auspices of the College of Life Sciences and Medicine, University of Aberdeen. All participants provided written informed consent and appropriate photographic release.

#### Participants

Ten participants (5 male/5 female) with a mean age of 31.0 years (*SD* = 9.9, Range = 23–55) were recruited from the University of Aberdeen’s School of Psychology.

#### Stimuli and Apparatus

The same smartphone was used as in Experiment 1. Each participant was asked to provide seven self-taken face photos (‘selfies’) to be used as their stimulus set. Consistent with the manufacturer’s instructions, we asked them to take these photos holding the phone at eye-level, in an adequately lit indoor location. We also requested that participants maintain a forward facing pose and a neutral expression.

**Fig 2:**
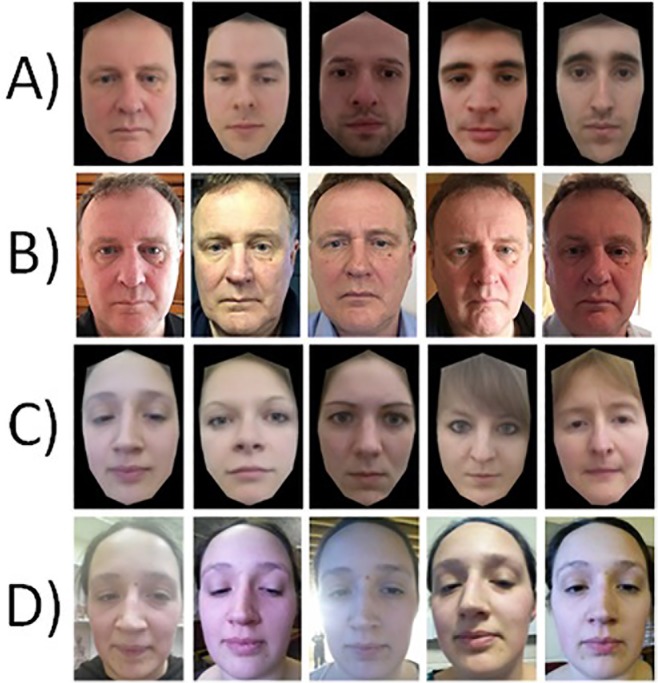
Example stimuli used in Experiment 2. Rows A and C shown the male and female face averages respectively. Rows B and D show five examples of the self-face-photos taken by the participants.

Participants took one ‘selfie’ per day for seven consecutive days, with each photo being taken in a different indoor location of their choice, on their own cell phones. All face images were then cropped to a size of 380 x 570 pixels. Each person’s set of seven photos were used to create their face average. Examples of the averages and individual images are shown in Fig. 2.

#### Design and Procedure

**Fig 3:**
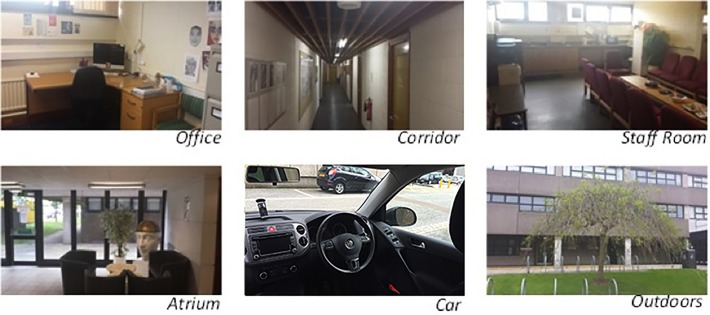
Images of the six different locations used in Experiment 2. Five indoor testing locations and one outdoor location.

The smartphone was encoded with a different target image of the participant on eight occasions using each of their seven individual ‘selfies’ and their face average. The order in which these eight images were encoded as targets was randomised across participants. At no point during the recognition trials did the individual know which of their face images had been encoded. On each of the eight occasions, participants were asked to try to unlock the phone using their face in six different locations (see Fig. 3). Participants were instructed to hold the phone at a comfortable distance from their face (ensuring all of their face was visible in the camera capture space), at which point they should press the power button on the device to activate the authentication program. The order of the locations was fixed (office, corridor, staff room, atrium, car, outdoors), with the eight testing cycles taking approximately 1 hour to complete.

### Results

**Fig 4:**
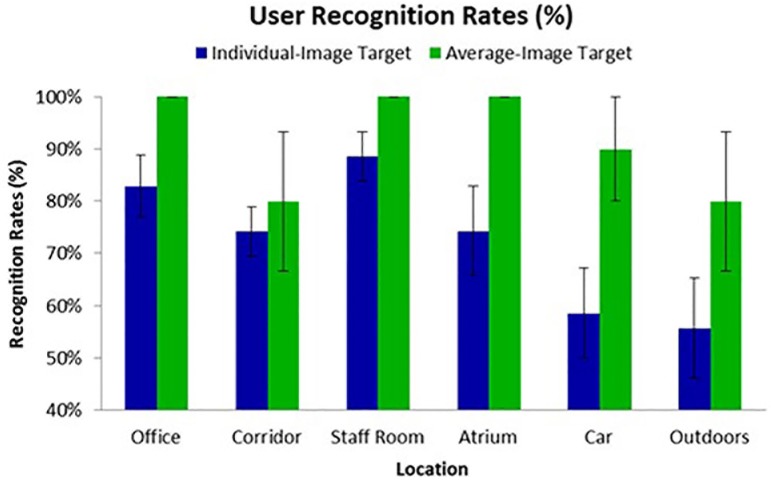
Mean user recognition rates for Experiment 2. Each participant was tested against seven different individual-image targets, and one average-image target, and these tests were repeated in each of the six locations. The figure shows mean performance by condition across all participants (*SD* in parentheses).

Fig. 4 shows mean recognition rates for individual photo targets and for average targets. In three locations, the average-image target performed perfectly (i.e. consistently at 100% accuracy, across all participants). A comparison pooled across all locations shows a highly significant advantage for average-image targets (Wilcoxon signed-rank test, two-tailed: *W* = 88, *p* < .001). Testing at individual locations showed significant advantages for average-images in Office, Atrium, Car and Outdoor locations (*W* = 0, 0, 1, 1 respectively, all *p*s < .05), while the numerical advantages in the Corridor and Staff Room locations failed to reach significance.

These results show a clear performance advantage for average targets over individual photos. Furthermore, this is consistent across locations. Overall, these findings extend the face average advantage to the recognition of the real smartphone users. Encoding a face average provides a significant and large benefit to the performance of these systems. It is interesting to note that the overall recognition rates are rather better in this setting than in Experiment 1. The results from standard indoor usage of the smartphone vary from about 50% to about 90%. However, in every case, the average faces do better. In fact, in three of our indoor environments, the office, staff room and atrium settings, the average elevates performance to perfect levels. This is quite an impressive jump, given that the averages are taken from 10 different people, and each average comprises only seven contributing images.

In general, a comparison across locations shows that the environments with good indoor lighting (Office, Staff Room, Atrium) out-perform outdoor or poorly-lit locations (e.g. Corridor—see Fig. 3). However, the average advantage holds very clearly for the more challenging outdoor setting. This is particularly impressive as the target images were all taken indoors (and the average is composed of these) while the test is performed outside. Outdoor performance with a single image is relatively low, as expected—and comparable only to the indoor in-car condition. However, the use of averages seems to go quite a long way to alleviating the well-known problems with outdoor face recognition, and provides the possibility that the system can reach a level of performance which makes it practical to use.

## Discussion

In two experiments we tested the recognition performance of the Samsung Galaxy face authentication system when it was encoded either with an individual face target or a face average target. We found significant average-face advantages in each experiment, despite the fact that the procedures (photographing screens vs real people) and settings (different physical locations and environmental lighting) were so widely varied. The improvement in authentication performance did not affect the system’s ability to reject images of an imposter (100% correct rejection rate in Experiment 1). In short, the use of averages improves user recognition in all settings, and in some (particularly outdoors) it renders the system practically useful, against a background of relatively poor baseline performance.

These findings extend the face average advantage reported by Jenkins and Burton [*19*] to another current and widely available commercial algorithm. Interestingly, while Jenkins and Burton used averages which were created from twenty photos of an individual, here we show that the effect can be replicated for real faces using an average created from just seven instances. Of course, we have tested only a single device here, the Samsung Galaxy smartphone. This seems a good candidate, since it is currently the world’s largest selling device which has an *in-built* face recognition system (programs with similar functionality are available for other phones, but for this model, the recognition system forms part of the core operating software). The fact that this provides a similar average-advantage to earlier tests using a different algorithm [*19*], suggests that this is a general property of face matching—though of course it will be necessary to examine this for particular cases. At the very least, our results suggest that developers should focus on the images used for matching, as well as the more traditional approach of focussing on matching algorithms themselves.

In fact, recent research has shown that developers are continuing to focus on improving the algorithm that matches a stored face to a novel image [*18*]. The Facebook affiliated DeepFace system [*24*] recently produced an accuracy rate of 97.25% when matching unconstrained photos, the highest yet recorded in a benchmark test. This system has been designed for face image recognition in social media contexts (e.g. automatically tagging people in uploaded photos) and its ability to reach similar levels of performance for real faces in everyday contexts has not been assessed. However, in line with previous systems which have approached such levels of accuracy on benchmark image matching tests, it is possible that it would produce a poorer level performance for real world face recognition (see Jenkins & Burton [*25*] for examples). In this paper, we demonstrate that, through no alterations to a commercial algorithm, we are able to improve the recognition of real faces in everyday conditions, simply by encoding a stable face representation.

It would be relatively straightforward to build this advantage directly into devices such as smartphones. We envisage that a new system might operate as follows: (i) Users would select seven digital images which provide an unobstructed view of their face. This is a relatively trivial requirement which could be accomplished through the user providing access to the image galleries in their Facebook or photo sharing applications; (ii) An automatic facial landmarking system would, for each image, mark the anatomical features required for averaging; (iii) Software would then create the person’s average and encode it as the stored representation of their face; (iv) On each occasion authentication occurs, an image of the user’s face would be captured and incorporated into their average. In this way, the average is being updated regularly, and will come implicitly to weight more recent photos more, while the updating procedure continues.

The only current constraint on the development of such a system is the lack of a reliable automatic face landmarking system. If the problem of automatic landmarking was resolved and the proposed system was implemented in smartphones, it could re-cast the face-based authentication system as a reliable security feature. Moreover, the fact that the effect was retained for outdoor recognition means that this method could also be applied to home and business site entry systems in which the user would be outdoors during the verification process.

Finally, we observe that the intention here is to reconcile what is known about human and automatic face recognition. Human perception of familiar faces is remarkably good—and remains much more robust across image conditions than even the best automatic recognition systems. Our approach here has been to incorporate a theory of human face familiarity into an automatic system. There may, of course, be better ways to do this. However, it does seem clear that automatic face recognition would benefit from an acknowledgement of the differences between familiar and unfamiliar faces, and from some emulation of familiar viewers.

## Supporting Information

S1 Table Experiment 1: User recognition accuracy for celebrity images. Mean recognition accuracy for the individual and average-image targets of each celebrity.
(DOCX)

S2 Table Experiment 1: Imposter rejection accuracy for celebrity images. Mean imposter rejection accuracy for the individual and average-image targets of each celebrity.
(DOCX)

S3 Table Experiment 2: User recognition accuracy for real faces. Mean recognition accuracy for the individual (Indiv.) and average-image (Avg.) targets for each participant and location.
(DOCX)

S1 File Celebrity identities and copyright information. Names of the celebrities whose images were used in Experiment 1 and license and attribution information for the individual instances of Hugh Jackman and Gwyneth Paltrow used in Fig. 1.
(DOCX)
