# Supplementary material for: Face Averages Enhance User Recognition for Smartphone Security
Source: PLoS One. 2015 Mar 25;10(3):e0119460. doi: 10.1371/journal.pone.0119460 (PMC4373928; doi:10.1371/journal.pone.0119460)
Supplement: S1 Table — Mean recognition accuracy for the individual and average-image targets of each celebrity. (DOCX) [file pone.0119460.s001.docx]

**Table S1. Experiment 1: User recognition accuracy for celebrity images.**

| **Celebrity ID** | **Encoded Image** | **Test Images** | **Recognition Accuracy %** |
| --- | --- | --- | --- |
| Tom Cruise | Average | Tom Cruise | 100% |
| Tom Cruise | Instances | Tom Cruise | 76% |
| Brad Pitt | Average | Brad Pitt | 100% |
| Brad Pitt | Instances | Brad Pitt | 52% |
| Tom Hanks | Average | Tom Hanks | 100% |
| Tom Hanks | Instances | Tom Hanks | 76% |
| Hugh Jackman | Average | Hugh Jackman | 20% |
| Hugh Jackman | Instances | Hugh Jackman | 20% |
| Matt Damon | Average | Matt Damon | 80% |
| Matt Damon | Instances | Matt Damon | 56% |
| Jodie Foster | Average | Jodie Foster | 100% |
| Jodie Foster | Instances | Jodie Foster | 44% |
| Gwyneth Paltrow | Average | Gwyneth Paltrow | 0% |
| Gwyneth Paltrow | Instances | Gwyneth Paltrow | 16% |
| Nicole Kidman | Average | Nicole Kidman | 80% |
| Nicole Kidman | Instances | Nicole Kidman | 52% |
| Kiera Knightley | Average | Kiera Knightley | 60% |
| Kiera Knightley | Instances | Kiera Knightley | 28% |
| Anne Hathaway | Average | Anne Hathaway | 40% |
| Anne Hathaway | Instances | Anne Hathaway | 28% |

*Note.* Mean recognition accuracy for the individual and average-image targets of each celebrity.
