# Supplementary material for: Face Averages Enhance User Recognition for Smartphone Security
Source: PLoS One. 2015 Mar 25;10(3):e0119460. doi: 10.1371/journal.pone.0119460 (PMC4373928; doi:10.1371/journal.pone.0119460)
Supplement: S2 Table — Mean imposter rejection accuracy for the individual and average-image targets of each celebrity. (DOCX) [file pone.0119460.s002.docx]

**Table S2. Experiment 1: Imposter rejection accuracy for celebrity images.**

| **Celebrity ID** | **Encoded Image** | **Test Images** | **Rejection Accuracy %** |
| --- | --- | --- | --- |
| Tom Cruise | Average | Brad Pitt | 100% |
| Tom Cruise | Instances | Brad Pitt | 100% |
| Brad Pitt | Average | Tom Hanks | 100% |
| Brad Pitt | Instances | Tom Hanks | 100% |
| Tom Hanks | Average | Hugh Jackman | 100% |
| Tom Hanks | Instances | Hugh Jackman | 100% |
| Hugh Jackman | Average | Matt Damon | 100% |
| Hugh Jackman | Instances | Matt Damon | 100% |
| Matt Damon | Average | Tom Cruise | 100% |
| Matt Damon | Instances | Tom Cruise | 100% |
| Jodie Foster | Average | Gwyneth Paltrow | 100% |
| Jodie Foster | Instances | Gwyneth Paltrow | 100% |
| Gwyneth Paltrow | Average | Nicole Kidman | 100% |
| Gwyneth Paltrow | Instances | Nicole Kidman | 100% |
| Nicole Kidman | Average | Kiera Knightley | 100% |
| Nicole Kidman | Instances | Kiera Knightley | 100% |
| Kiera Knightley | Average | Anne Hathaway | 100% |
| Kiera Knightley | Instances | Anne Hathaway | 100% |
| Anne Hathaway | Average | Jodie Foster | 100% |
| Anne Hathaway | Instances | Jodie Foster | 100% |

*Note.* Mean imposter rejection accuracy for the individual and average-image targets of each celebrity.
