# Supplementary material for: Face Averages Enhance User Recognition for Smartphone Security
Source: PLoS One. 2015 Mar 25;10(3):e0119460. doi: 10.1371/journal.pone.0119460 (PMC4373928; doi:10.1371/journal.pone.0119460)
Supplement: S3 Table — Mean recognition accuracy for the individual (Indiv.) and average-image (Avg.) targets for each participant and location. (DOCX) [file pone.0119460.s003.docx]

**Table S3. Experiment 2: User recognition accuracy for real faces.**

| **Participant** | **Office** | | **Corridor** | | **Staff Room** | | **Atrium** | | **Car** | | **Outdoors** | |
| --- | --- | --- | --- | --- | --- | --- | --- | --- | --- | --- | --- | --- |
|  | **Avg.** | **Indiv.** | **Avg.** | **Indiv.** | **Avg.** | **Indiv.** | **Avg.** | **Indiv.** | **Avg.** | **Indiv.** | **Avg.** | **Indiv.** |
| P01 | 100.0% | 100.0% | 100.0% | 57.1% | 100.0% | 100.0% | 100.0% | 85.7% | 100.0% | 85.7% | 100.0% | 85.7% |
| P02 | 100.0% | 85.7% | 100.0% | 85.7% | 100.0% | 85.7% | 100.0% | 57.1% | 100.0% | 57.1% | 100.0% | 71.4% |
| P03 | 100.0% | 85.7% | 100.0% | 85.7% | 100.0% | 100.0% | 100.0% | 100.0% | 100.0% | 14.3% | 100.0% | 71.4% |
| P04 | 100.0% | 100.0% | 100.0% | 71.4% | 100.0% | 100.0% | 100.0% | 85.7% | 100.0% | 85.7% | 0.0% | 0.0% |
| P05 | 100.0% | 85.7% | 100.0% | 71.4% | 100.0% | 71.4% | 100.0% | 71.4% | 100.0% | 57.1% | 100.0% | 42.9% |
| P06 | 100.0% | 57.1% | 0.0% | 42.9% | 100.0% | 85.7% | 100.0% | 85.7% | 100.0% | 71.4% | 100.0% | 42.9% |
| P07 | 100.0% | 85.7% | 100.0% | 85.7% | 100.0% | 100.0% | 100.0% | 71.4% | 100.0% | 42.9% | 100.0% | 85.7% |
| P08 | 100.0% | 100.0% | 100.0% | 85.7% | 100.0% | 100.0% | 100.0% | 71.4% | 100.0% | 71.4% | 100.0% | 85.7% |
| P09 | 100.0% | 85.7% | 0.0% | 71.4% | 100.0% | 85.7% | 100.0% | 42.9% | 100.0% | 85.7% | 0.0% | 14.3% |
| P10 | 100.0% | 42.9% | 100.0% | 85.7% | 100.0% | 57.1% | 100.0% | 71.4% | 0.0% | 14.3% | 100.0% | 57.1% |

*Note*. Mean recognition accuracy for the individual (Indiv.) and average-image (Avg.) targets for each participant and location.
