# Supplementary material for: Face Averages Enhance User Recognition for Smartphone Security
Source: PLoS One. 2015 Mar 25;10(3):e0119460. doi: 10.1371/journal.pone.0119460 (PMC4373928; doi:10.1371/journal.pone.0119460)
Supplement: S1 File — Names of the celebrities whose images were used in Experiment 1 and license and attribution information for the individual instances of Hugh Jackman and Gwyneth Paltrow used in Fig. 1. (DOCX) [file pone.0119460.s004.docx]

**File S1. Celebrity identities and copyright information.** Names of the celebrities whose images were used in Experiment 1 and license and attribution information for the individual instances of Hugh Jackman and Gwyneth Paltrow used in Figure 1.

**Figure 1: Celebrity Identities**

**Males** (L-R): Hugh Jackman, Brad Pitt, Tom Cruise, Tom Hanks, Matt Damon.

**Females**(L-R): Gwyneth Paltrow, Anne Hathaway, Jodie Foster, Keira Knightley and Nicole Kidman.

**Figure 1: Licence and Attribution Information**

The five images of Hugh Jackman (HJ) and Gwyneth Paltrow (GP) used in Figure 1 were selected from a Google Image search using the ‘User Rights’ filter set at ‘Free to reuse’. Each of the images was labelled with a CC BY-SA 2.0 Generic or a CC BY-SA 3.0 Unported Creative Commons licence. Individual licence and attribution is outlined below.

**HJ Image 1:** Hugh Jackman in 2012, Photographs by Eva Rinaldi.

**Licence:** CC BY-SA 2.0. Generic

**Downloaded from:**

http://ro.wikipedia.org/wiki/Hugh_Jackman#mediaviewer/Fi%C8%99ier:Hugh_Jackman_4,_2012.jpg

**HJ Image 2:** Hugh Jackman Apr 09b, uploaded by Gryffindor to Wikipedia April 27th 2009.

**Licence:** CC BY-SA 3.0 Unported

**Downloaded from:**

http://en.wikipedia.org/wiki/Hugh_Jackman#mediaviewer/File:HughJackmanApr09b.jpg

**HJ Image 3:** Hugh Jackman in 2012, Photographs by Eva Rinaldi.

**Licence:** CC BY-SA 2.0 Generic

**Downloaded from:**

http://commons.wikimedia.org/wiki/File:Hugh_Jackman_-_Flickr_-_Eva_Rinaldi_Celebrity_and_Live_Music_Photographer_(4).jpg#filelinks

**HJ Image 4:** HughJackmanByPaulCush2011, Photographs by Paul Cush

**Licence:** CC BY-SA 3.0 Unported

**Downloaded from:**

http://fr.wikipedia.org/wiki/Hugh_Jackman#mediaviewer/Fichier:HughJackmanByPaulCush2011.jpg

**HJ Image 5:** Hugh Jackman by Gage Skidmore, Photographs by Gage Skidmore

**Licence:** CC BY-SA 3.0 Unported

**Downloaded from:**

http://de.wikipedia.org/wiki/Hugh_Jackman#mediaviewer/Datei:Hugh_Jackman_by_Gage_Skidmore.jpg

**GP Image 1:** Gwyneth Paltrow at the Hollywood Walk of Fame ceremony, photograph by Richard Yaussi

**Licence:** CC BY-SA 2.0 Generic

**Downloaded from:**

http://commons.wikimedia.org/wiki/File:Gwyneth_Paltrow_at_the_Hollywood_Walk_of_Fame_ceremony_-_20101213.jpg

**GP Image 2:** Gwyneth Paltrow 2010 by Romina Espinosa

**Licence:** CC BY-SA 3.0 Unported

**Downloaded from:**

http://commons.wikimedia.org/wiki/File:Gwyneth_Paltrow_2010.jpg

**GP Image 3:** Gwyneth Paltrow Iron Man 3 avp Paris, Images by Georges Biard

**Licence:** CC BY-SA 3.0 Unported

**Downloaded from:**

http://creativecommons.org/licenses/by-sa/3.0/

**GP Image 4:** Face of actress Gwyneth Paltrow, Author Jared Purdy

**Licence:** CC BY-SA 3.0 Unported

**Downloaded from:**

http://commons.wikimedia.org/wiki/File:Gwyneth_Paltrow_face.jpg

**GP Image 5:** Gwyneth Paltrow By Andrea Raffin 2011, uploaded to Wikipedia by Electroguv on September 1st 2011

**Licence:** CC BY-SA 3.0 Unported

**Downloaded from:**

http://en.wikipedia.org/wiki/Gwyneth_Paltrow#mediaviewer/File:GwynethPaltrowByAndreaRaffin2011.jpg
